# Developmental changes rather than repeated administration drive paracetamol glucuronidation in neonates and infants

**DOI:** 10.1007/s00228-015-1887-y

**Published:** 2015-07-03

**Authors:** Elke H. J. Krekels, Saskia van Ham, Karel Allegaert, Jan de Hoon, Dick Tibboel, Meindert Danhof, Catherijne A. J. Knibbe

**Affiliations:** Division of Pharmacology, Leiden Academic Center for Drug Research, Leiden, The Netherlands; Department of Development and Regeneration, KU Leuven, Leuven, Belgium; Neonatal Intensive Care Unit, University Hospitals, Leuven, Belgium; Center for Clinical Pharmacology, University Hospitals, Leuven, Belgium; Intensive Care and Department of Pediatric Surgery, Erasmus MC-Sophia Children’s Hospital, Rotterdam, The Netherlands; Department of Clinical Pharmacy, St. Antonius Hospital, PO Box 2500, 3430 EM Nieuwegein, The Netherlands

**Keywords:** Paracetamol, Glucuronidation, Multiple dosing, Paediatrics

## Abstract

**Purpose:**

Based on recovered metabolite ratios in urine, it has been concluded that paracetamol glucuronidation may be up-regulated upon multiple dosing. This study investigates paracetamol clearance in neonates and infants after single and multiple dosing using a population modelling approach.

**Methods:**

A population pharmacokinetic model was developed in NONMEM VI, based on paracetamol plasma concentrations from 54 preterm and term neonates and infants, and on paracetamol, paracetamol-glucuronide and paracetamol-sulphate amounts in urine from 22 of these patients. Patients received either a single intravenous propacetamol dose or up to 12 repeated doses.

**Results:**

Paracetamol and metabolite disposition was best described with one-compartment models. The formation clearance of paracetamol-sulphate was 1.46 mL/min/kg^1.4^, which was about 5.5 times higher than the formation clearance of the glucuronide of 0.266 mL/min/kg. The renal excretion rate constants of both metabolites was estimated to be 11.4 times higher than the excretion rate constant of unchanged paracetamol, yielding values of 0.580 mL/min/kg. Developmental changes were best described by bodyweight in linear relationships on the distribution volumes, the formation of paracetamol-glucuronide and the unchanged excretion of paracetamol, and in an exponential relationship on the formation of paracetamol-sulphate. There was no evidence for up-regulation or other time-varying changes in any of the model parameters. Simulations with this model illustrate how paracetamol-glucuronide recovery in urine increases over time due to the slower formation of this metabolite and in the absence of up-regulation.

**Conclusions:**

Developmental changes, described by bodyweight-based functions, rather than up-regulation, explain developmental changes in paracetamol disposition in neonates and infants.

**Electronic supplementary material:**

The online version of this article (doi:10.1007/s00228-015-1887-y) contains supplementary material, which is available to authorized users.

## Introduction

Paracetamol (acetaminophen) is a commonly prescribed drug to treat moderate pain or fever in adults and children, including neonates. Paracetamol was also shown to reduce morphine requirements in post-operative neonates and infants [1], and an association between paracetamol exposure and closure of a patent ductus arteriosus in neonates has also been suggested [2–4].

In adults, the main elimination pathway for paracetamol is glucuronidation. Since the glucuronidation capacity is greatly reduced in neonates, the elimination of paracetamol is directed towards sulphation and unchanged renal excretion in these patients [5]. Different developmental patterns for the various clearance pathways of paracetamol result in continuous changes in the relative contribution of each pathway to the overall paracetamol clearance as well as in changes in total paracetamol clearance. Although these changes are generally thought to be the main cause of age-dependent differences of drug dose requirements in children [5], the influence of developmental changes on the pharmacokinetics of paracetamol and its metabolites have not been determined. Moreover, there have been reports of the glucuronidation of paracetamol being up-regulated upon multiple dosing in adults [6] as well as in neonates and infants [7, 8]. These studies were all based on recovered paracetamol and metabolite ratios in urine, but these results have never been confirmed in a population analysis. One of the advantages of a population analysis is that elimination pathways and the demographic and clinical factors affecting them can be studied separately [9].

The current study investigates to what extent paracetamol is cleared through glucuronidation, sulphation or unchanged renal excretion upon single and multiple dosing in neonates and infants, using paracetamol plasma concentrations and recovered paracetamol and metabolite amounts in urine. This is done by firstly quantifying the influence of developmental changes on the pharmacokinetics of paracetamol after intravenous administration of propacetamol, which is the precursor of paracetamol, and subsequently investigating potential time-related trends in paracetamol glucuronidation.

## Methods

The current analysis was performed based on pooled data of plasma paracetamol concentrations and recovered paracetamol and metabolite amounts in urine of earlier reported studies on intravenous propacetamol disposition in preterm and term neonates and infants [7, 10, 11]. Details on patient characteristics, inclusion criteria and sampling are available in the original references. Study protocols were approved by the local ethics board of the University Hospital Leuven, and patients were included after written informed consent was obtained from the parents or legal guardians of patients included in the study.

### Plasma paracetamol pharmacokinetic studies

Neonates exposed to either single or repeated intravenous administration of propacetamol were included.

In the single-dose study, neonates received one dose of 20 or 40 mg/kg of propacetamol (paracetamol equivalent 10 or 20 mg/kg) on the first day of postnatal life, and plasma samples were collected up to 10 h afterwards. Propacetamol was administered when neonates underwent minor painful procedures or as additional treatment in neonates on opioid treatment. Paracetamol plasma concentrations were determined using fluorescence polarisation immunoassay (Adx, Abbott Laboratories, North Chicago, IL). Lower limit of quantification was 1 mg/L, and precision was 7 % [10].

In the repeated dose study, a loading dose of 30 mg/kg propacetamol was administered, followed by at least 1, and up to 11 (median = 5) maintenance doses of 20 mg/kg (paracetamol equivalent 15 and 10 mg/kg, respectively), using a postmenstrual age (PMA) dependent dosing interval of q6h to q12h. Inclusion criteria were similar to the single-dose study, but the age of the patients was not limited to the first day of postnatal life. Paracetamol plasma concentrations were measured using high-performance liquid chromatography (HPLC) as described earlier [11]. The lower limit of quantification was 0.08 mg/L, and variation coefficients of intra- and inter-day accuracy and precision were all below 15 %.

In these studies, metabolite concentrations were not analysed in plasma.

### Urine paracetamol metabolite study

Urine collections were performed in neonates exposed to repeated dose administration of propacetamol. Collection was started at initiation of the loading dose, and all urine was collected in 6, 8, or 12 h aliquots, related to the PMA-dependent dosing interval. At the end of every collection time interval, total urine volume was recorded and a representative urine sample was stored until analysis. Urine collection was stopped when the latest collection period was incomplete. At best, urine collection was stopped one time interval after the last administration. Paracetamol-glucuronide, paracetamol-sulphate and unchanged paracetamol urine concentrations were determined using HPLC methodology as described earlier [7]. The lower limit of quantification was 1 μg/L. Variation coefficients in intra- and inter-day accuracy and precision were all below 15 %.

### Pharmacokinetic analysis

A population pharmacokinetic model was developed using NONMEM VI (Globomax LLC, Hanover, MD, USA) with PLT Tools [12] for visualization of the data. The first-order estimation algorithm with interaction (FOCE-I) was used. Propacetamol doses and the recovered metabolite amounts in urine were expressed as paracetamol equivalents.

Model development was performed in four steps: (1) choice of the structural model, (2) choice of the error model, (3) covariate analysis and (4) evaluation of the model. For the inclusion of fixed and random model parameters as well as covariates, a decrease in objective function corresponding to *p* < 0.01 was considered to be statistically significant assuming a *χ*^2^ distribution. In addition, the following goodness-of-fit plots were used for diagnostic purposes: (1) observed versus population predictions, (2) observed versus individual predictions, (3) conditional weighted residuals versus time and (4) conditional weighted residuals versus population predictions.

For the structural model, one and two compartments were explored to describe the time-course of the paracetamol plasma concentrations. Since the metabolite concentrations in plasma were not measured, assumptions for the structural model were necessary, for which the same approach was followed as previously reported population model for paracetamol [13]. Briefly, the distribution volumes of both metabolites were set to 18 % of the volume of the parent compound, based on reported values in adults [14]. Based on physiological plausibility, the same excretion rate constant (*k*) was assumed for both metabolites, and this value was estimated to be a multiple of the excretion rate constant of the parent compound. Different values were estimated for the formation rate constants of the metabolites.

Inter-individual variability in the model parameters was tested assuming log-normal distribution using Eq. 1:

1 $$ {P}_i={P}_p\;*\; \exp\;\left({\eta}_i\right) $$

in which *P*_*i*_ is the individual parameter estimate for the *i*th individual, *P*_*p*_ represents the population parameter value (also known as typical parameter value) for parameter *P*, and *η*_*i*_ is a random variable for the *i*th individual from a normal distribution with a mean of zero and an estimated variance. Residual unexplained variability in the observed drug and metabolite concentrations proportional, additive and a combination error model were tested.

A systematic covariate analysis was performed to identify the best descriptor of the developmental changes in the pharmacokinetics of paracetamol and its metabolites. The covariates bodyweight, postnatal age (PNA) and PMA were tested in linear and exponential equations:

2 $$ {P}_i={P}_p\;*\;{(cov)}^n $$

in which *P*_*i*_ and *P*_*p*_ represent the individual and population parameter values respectively and *cov* represents the value of the covariate for the *i*th individual. *n* is the exponent that is fixed to 1 in linear equations and estimated in exponential equations. Additionally, sex, preterm birth and the clinical trial that data were obtained from were tested as categorical covariates, estimating parameter values for one subpopulation as a fraction of the other.

All covariates were added to the base model separately, and considered statistically significant if the objective function decreased 6.63 points or more (*p* < 0.01). When more than one significant covariate was found for the base model, the covariate-adjusted model with the largest decrease in objective function was chosen as the basis to explore the influence of additional covariates with the use of the same criteria. Afterwards, a backwards deletion procedure was performed on the full model, in which the included covariates were excluded one at a time and only retained if the increase in objective function was more than 7.88 points (corresponding to *p* < 0.005).

The presence of time-related changes in the formation of paracetamol-glucuronide (i.e. up-regulation) was further investigated by either estimating the formation clearance of the paracetamol-glucuronide for later doses within an individual as a multiple of the formation clearance of the first dose, or by estimating continuous linear or exponential changes in the glucuronidation clearance over time. The criteria used for the covariate model building were also used to determine whether inclusion of time-related changes for paracetamol glucuronidation in the model significantly improved the model fit to the data.

### Model evaluation

Identifiability of model parameters and model stability were investigated using the condition number and a bootstrap procedure. To obtain the condition number, the highest eigenvalue in the NONMEM output of the final model fit was divided by the lowest eigenvalue. In the bootstrap procedure, 100 replicates of the original data were generated by resampling with replacement, and these datasets were fit to the final model.

In addition, goodness-of-fit plots were generated, including plots of both individual predicted and population predicted concentrations (in plasma) or amounts (in urine) versus observed concentrations or amounts. Individual predictions are the result of a model fit to the data, which is based on dosing history, sampling time, individual covariate information and observed concentrations. Population predictions on the other hand are based on the population parameter values, the dosing history, sampling times and individual covariate information alone. Plots of conditional weighted residuals versus time and versus population predicted concentrations were also generated, and special attention was paid to the former to investigate potential time-trends.

Finally, normalized prediction distribution errors (NPDE) [15] were used as a simulation-based diagnostic to assess model performance. For this, the dataset was simulated 500 times. The NPDE package in R was subsequently used to calculate the NPDE for each observation, by comparing the observed concentrations and amounts to the simulated reference distribution. Special attention was again paid to the plot of NPDE’s versus time, to further establish whether there were any time-related trends in the observed paracetamol concentrations or recovered paracetamol and metabolite amounts in urine.

### Simulations

To determine the recovery pattern of paracetamol and its metabolites in urine over time, model simulations were performed with the final model and a ‘typical individual’, which is defined as a hypothetical individual with population values (i.e. typical values) for all model parameters. The bodyweight of this ‘typical individual’ was selected to be 2.5 kg, which is the mean bodyweight in the dataset. In the simulation, a paracetamol equivalent dose of 15 mg/kg was administered intravenously during a 15-min infusion, analogue to the studies from which the data analysed in the current study were obtained. The simulated relative cumulative amount of paracetamol, paracetamol-glucuronide and paracetamol-sulphate recovered in urine within the first 36 h was determined. Additionally, the amount of paracetamol, paracetamol-glucuronide and paracetamol-sulphate recovered within each 6-h urine collection interval was determined.

## Results

This analysis was based on data from 54 patients, who at the start of the study had a median bodyweight of 2.5 kg (range, 0.5–6.3 kg), a median PNA of 1 day (range, 1–140 days) and a median PMA of 36 weeks (range, 27–60 weeks). A total of 353 observed paracetamol plasma concentrations were available for analysis and 435 observations of the amounts of the parent compound or the metabolites in urine samples. The median number of propacetamol doses per patient was 1 (range, 1–12).

**Fig. 1:**
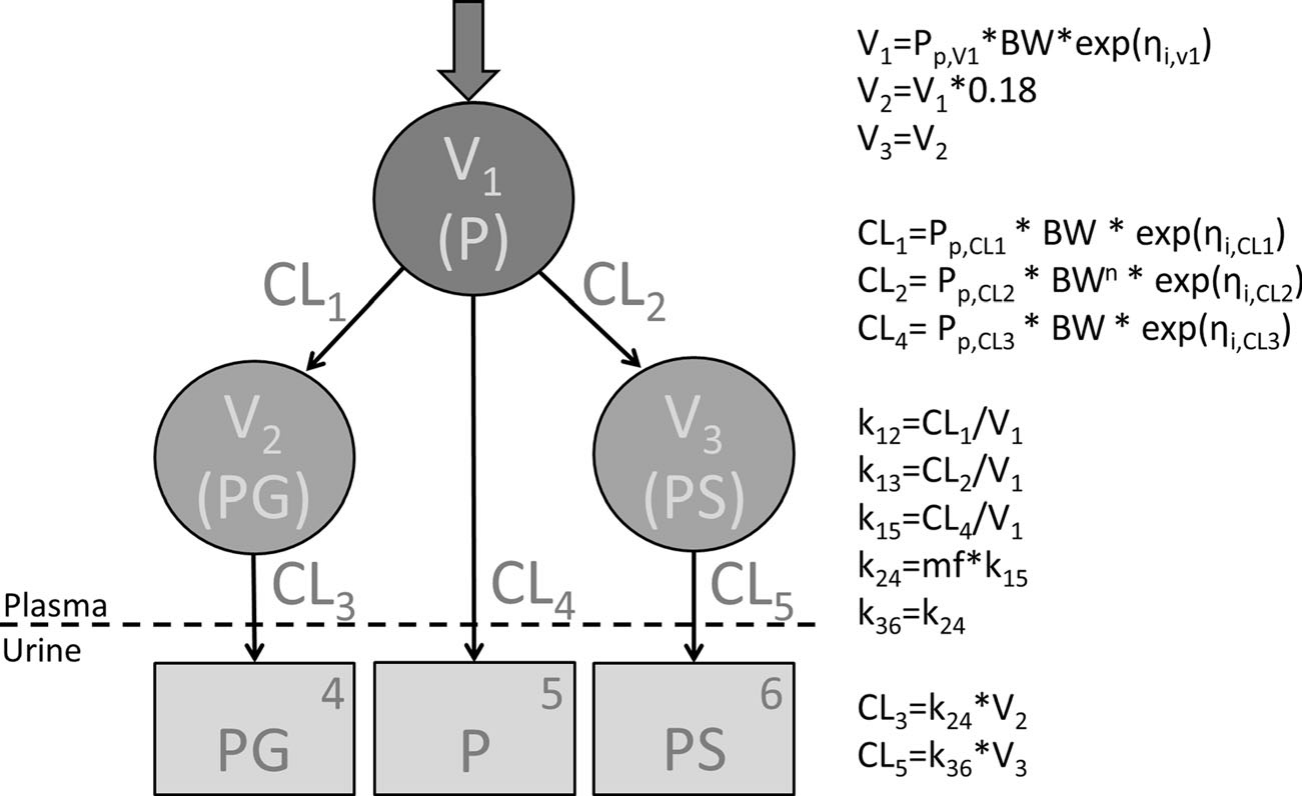
Schematic representation of the pharmacokinetic model for paracetamol and two of its metabolites and the equations used for each model parameter. *V* distribution volume of the designated compartment, *CL* clearance of the designated pathway, *P* paracetamol, *PG* paracetamol-glucuronide, *PS* paracetamol-sulphate, *P*_*p*_ population parameter value of the designated parameter, *BW* bodyweight, *η* random variable from a normal distribution with a mean of 0 and estimated variance, *n* exponent, *k* elimination rate constant of the designated elimination route, *mf* multiplication factor

Figure 1 depicts a schematic representation of the pharmacokinetic model for paracetamol and its metabolites, as well as the equations used to describe the parameters in the model.

Inter-individual variability was identified for the distribution volume of paracetamol, for the formation clearances of the metabolites and for the unchanged renal clearance of paracetamol. For the intra-individual variability and residual error, a combination model was most suitable for the paracetamol concentrations in plasma, while additive errors were best for the recovered amounts of parent compound and metabolites in urine.

Bodyweight in a linear equation was found to be the most predictive covariate for the distribution volume of paracetamol (V_1_) and as the distribution volumes of the metabolites were a percentage of this distribution volume, the covariate relationship perpetuated in these parameters as well. Additionally, bodyweight was found to be the most predictive covariate for the formation of paracetamol-glucuronide (CL_1_) and the unchanged excretion of paracetamol (CL_4_) in a linear relationship, with the latter was also being perpetuated to the excretion clearance of the metabolites (CL_3_ and CL_5_). For the formation of paracetamol-sulphate (CL_2_), the most predictive covariate relationship was bodyweight in an exponential equation with an estimated exponent of 1.40 (relative standard error, 9.3 %).

After inclusion of the bodyweight-based covariate relationships, no other statistically significant covariates related to either patient characteristics or clinical trial characteristics could be identified. Diagnostics of this model did not suggest time-related model misspecification. Additionally, when estimating the value for glucuronidation clearance (CL_1_) for the later doses as a multiple of the glucuronidation clearance value for the first dose, the estimated value of this fraction was 1.04, which did not statistically significantly improve the model fit. Including continuous time-related changes on this parameter in a linear or exponential relationships yielded estimates of respectively the slope and exponent that approached 0 and no statistically significant improvement of the model fit. All these results indicate that the glucuronidation clearance (CL_1_) at earlier and later time points remain essentially the same.

Table 1 shows the parameter values obtained in the final model fit as well as the mean parameter values obtained in the bootstrap procedure. The coefficients of variations for the structural parameters are all below 30 % in both the original model fit as well as in the bootstrap procedure. This indicates that these parameters could be estimated with good precision from the data. The fact that all mean parameter values in the bootstrap procedure are within 10 % of the values obtained in the original model fit indicates that the model is robust. The condition number of this model was 38, with a condition number lower than 1000 indicating a model to be sufficiently supported by the data.

**Table 1:** Obtained pharmacokinetic model parameters including the relative standard error (RSE) of the original model fit and of the bootstrap

Parameter (unit)	Model fit (RSE%)	Bootstrap (RSE%)
Fixed effects		
V_1_ (L/kg)	1.06 (4.34)	1.05 (4.47)
V_2_ = V_3_ (L/kg)	0.191 (derived parameter)	0.190 (derived parameter)
CL_1_ (mL/min/kg)	0.266 (17.4)	0.273 (18.7)
CL_2_ (mL/min/kg^n^)	1.46 (14.8)	1.45 (15.9)
CL_4_ (mL/min/kg)	0.285 (6.98)	0.282 (7.99)
* n*	1.40 (9.29)	1.41 (9.52)
mf	11.3 (21.5)	11.3 (23.3)
CL_3_ = CL_5_ (mL/min/kg)	0.580 (derived parameter)	0.577 (derived parameter)
Inter-individual variability (variance)		
V_1_	0.0925 (29.5)	0.0889 (32.7)
CL_1_	0.599 (41.6)	0.561 (44.7)
CL_2_	0.312 (33.3)	0.294 (32.7)
CL_4_	0.0879 (52.2)	0.0936 (74.2)
Residual variability (variance)		
P plasma, additive (mg/L)	0.354 (29.4)	0.383 (42.7)
P plasma, proportional	0.0198 (16.5)	0.0188 (30.7)
PG urine, additive (mg)	0.223 (14.5)	0.222 (18.6)
P urine , additive (mg)	0.188 (27.5)	0.183 (14.5)
PS urine, additive (mg)	0.332 (35.9)	0.341 (29.6)

See Fig. 1 for the explanation of the symbols

**Fig. 2:**
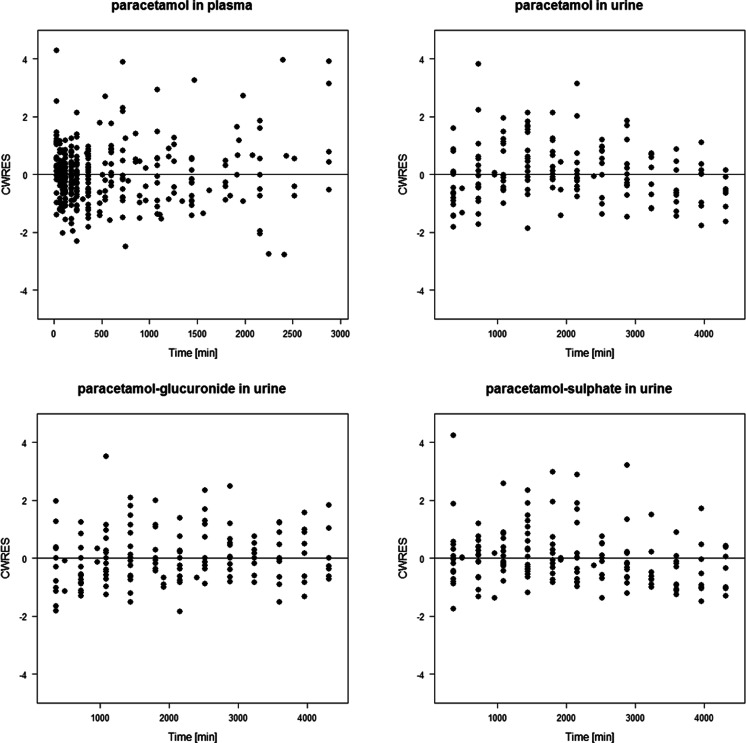
Conditional weighted residuals versus time for paracetamol concentrations in plasma, and for the recovered amounts of paracetamol-glucuronide, unchanged paracetamol and paracetamol-sulphate in urine

The plot of conditional weighted residuals versus time presented in Fig. 2 confirms that there is no time-related model misspecification of paracetamol plasma concentrations, or of the recovered paracetamol and metabolite amounts in urine. The plots of individual prediction versus observed values in supplemental Fig. S1A show no bias and neither do plots of conditional weighted residuals versus the predicted concentration in supplemental Fig. S1C. Both plots suggest that throughout the study, paracetamol concentrations in plasma as well as paracetamol and metabolite amounts in urine can be accurately described by a pharmacokinetic model that does not include time-varying elements in the glucuronidation clearance of paracetamol (CL_1_) or any other model parameter.

The plots of the population predictions versus observed concentrations in supplemental Fig. S1B show small trends, especially for the metabolites. This suggests that although description of the data with this model is accurate (supplemental Fig. S1A), caution is warranted in using this model for predictions outside the studied patient population or in basing dosing algorithms on this model.

The NPDE analysis showed that the final model can accurately predict paracetamol concentrations in plasma and paracetamol and metabolite amounts in urine for this dataset. Supplemental Fig. 2 show the distribution of NPDE’s in time. These unbiased plots further confirm our finding that in this young population, paracetamol elimination can be described by a model without time-varying aspects in any of the studied clearance routes. Expected means and variance values for the NPDE distribution of accurate models are 0 and 1, respectively. The values for the observed data deviated slightly from these values with 0.128 and 1.03 respectively for paracetamol in plasma, −0.128 and 0.870 for paracetamol in urine, 0.0777 and 1.10 for paracetamol-glucuronide in urine and 0.0925 and 0.822 for paracetamol-sulphate in urine.

**Fig. 3:**
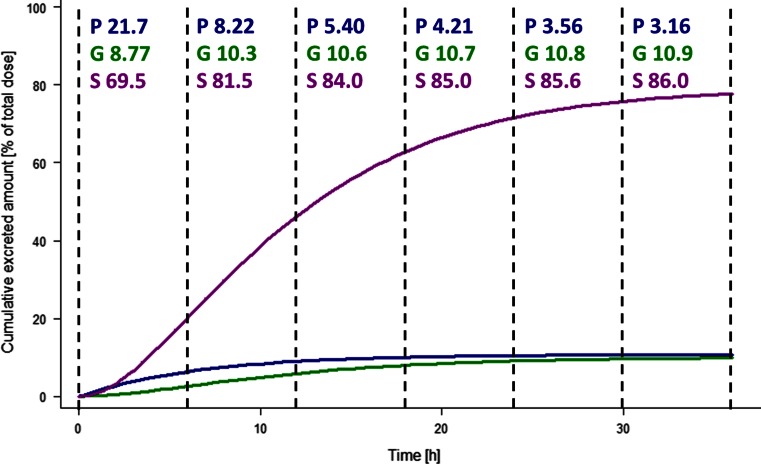
Relative cumulative amount of paracetamol-glucuronide (*green*, G), unchanged paracetamol (*blue*, P) and paracetamol-sulphate (*purple*, S) recovered in urine after a single dose in a typical individual with a bodyweight of 2.5 kg, with the recovered metabolite amounts being expressed in molar paracetamol equivalents. Percentage paracetamol and metabolites recovered in each 6 h urine collection interval are indicated at the top of the graph

The results of the simulation procedure with the final model in Fig. 3 illustrate how the percentage of paracetamol and metabolites that is recovered in urine in consecutive 6-h intervals changes over time. Although all clearances in the model remain constant, the percentage of the recovered paracetamol-glucuronide increases over time. This can be explained by the slower formation of the glucuronide compared to the sulphate, which causes the recovery of glucuronide in urine to occur later in time.

## Discussion

In this study, a population pharmacokinetic model was developed to describe the time-course of paracetamol and two of its common metabolites in preterm and term neonates and infants. In order to investigate potential up-regulation of paracetamol glucuronidation upon multiple dosing in these patients, the influence of developmental changes on the disposition of paracetamol was first quantified. Subsequently, we investigated whether the relative contributions of the various clearance pathways remained constant upon multiple dosing or whether there was up-regulation of the glucuronidation pathway in particular. No time-related factors other than changes in bodyweight reflecting developmental changes could be identified to influence the elimination of paracetamol upon single and multiple dosing. The thorough model evaluation and validation procedures applied to the pharmacokinetic model support the conclusions from this study.

Developmental changes in drug clearance are thought to be the leading cause of age-dependent differences in drug requirements in the paediatric population [5]. According to this model, paracetamol is mostly metabolized by sulphation in neonates and infants, which is an efficient elimination pathway at birth [5]. The exponential covariate relationship with bodyweight that was found to describe the development of this pathway in the current study population, suggests that this pathway is developing rapidly. The current analysis shows the glucuronidation capacity to increase with bodyweight as well, although in this very young population, the increase in glucuronidation capacity is still slower than the increase in sulphation capacity. The increase in the renal excretion of unchanged paracetamol and of the two paracetamol metabolites is similar to the increase in glucuronidation capacity. Most of this excretion occurs through glomerular filtration, which is blood flow dependent.

Both renal excretion and drug glucuronidation are known to reach full maturity well after the neonatal period [5]. As the capacity of sulphotransferases is limited, the glucuronidation pathway increases in importance throughout childhood, causing sulphation to be a relatively minor pathway for paracetamol clearance in adults. Determining the full developmental patterns for these three elimination pathways of paracetamol throughout childhood was outside the scope of this study.

Studies by our own group and by others, in both adult and paediatric populations have previously been interpreted to suggest that the glucuronidation clearance of paracetamol is up-regulated upon multiple dosing [6–8]. These studies, however, relied on analysing the ratios of paracetamol and metabolites recovered in urine over consecutive collection-intervals and did not take paracetamol plasma concentrations into account. We were able to obtain a comprehensive understanding of the underlying metabolic and elimination processes of paracetamol, by combining pharmacokinetic data from plasma and urine in a population modelling approach. Figure 3 illustrates that the expected ratio of paracetamol and the two metabolites recovered over 6-h intervals in urine is indeed continuously changing, despite the fact that all clearances remain constant over time. As the formation of the glucuronide is considerably slower than the formation of the sulphate metabolite, the glucuronide metabolite is recovered in urine later. Although in line with previous observations that the fraction of recovered paracetamol-glucuronide increases during the first days of paracetamol treatment, the previous conclusion that this is caused by up-regulation of the glucuronidation pathway should be rejected. Therefore, even though data on complete urine recovery can be used to investigate which fractions of drugs are eliminated through various pathways, in our opinion, methods based on recovered drug and metabolite ratios should not be used to study time-varying changes in drug elimination pathways, either in adults or in paediatrics. We recommend confirming these findings with a population analysis instead.

The necessary assumption in the structural model regarding pharmacokinetic parameters of the metabolites is not expected to influence our conclusions on the developmental patterns of elimination pathways or on the absence of up-regulation of the glucuronidation pathway in particular. Our assumptions were based on literature findings [13, 14] and as all data points are influenced similarly by these assumptions, they are not expected to have introduced systematic trends or bias related to time after start of treatment or related to developmental difference between patients.

In conclusion, there is no evidence for up-regulation of paracetamol glucuronidation in preterm and term neonates and infants. The only increases in paracetamol glucuronidation that could be identified in this population were resulting from growth and developmental changes, and these were adequately described by bodyweight-based covariate relationships.

## Electronic supplementary material

Supp fig. 1 Goodness-of-fit plots for the final model. **a** Individual predicted versus observed plots, **b** population predicted versus observed plots, **c** Conditional weighted residuals versus predicted concentration high resolution image (PNG 143 kb) (PNG 155 kb) High resolution image (TIFF 199 kb)

(GIF 125 kb)

Supp fig. 2 Normalized prediction distribution errors versus time for paracetamol concentrations in plasma, and for the recovered amounts of paracetamol-glucuronide, unchanged paracetamol, and paracetamol-sulphate in urine high resolution image (GIF 266 kb)

High resolution image (TIFF 760 kb)
