# Supplementary figures and images for: Developmental changes rather than repeated administration drive paracetamol glucuronidation in neonates and infants
Source: Eur J Clin Pharmacol. 2015 Jul 3;71(9):1075–82. doi: 10.1007/s00228-015-1887-y (PMC4532713; doi:10.1007/s00228-015-1887-y)

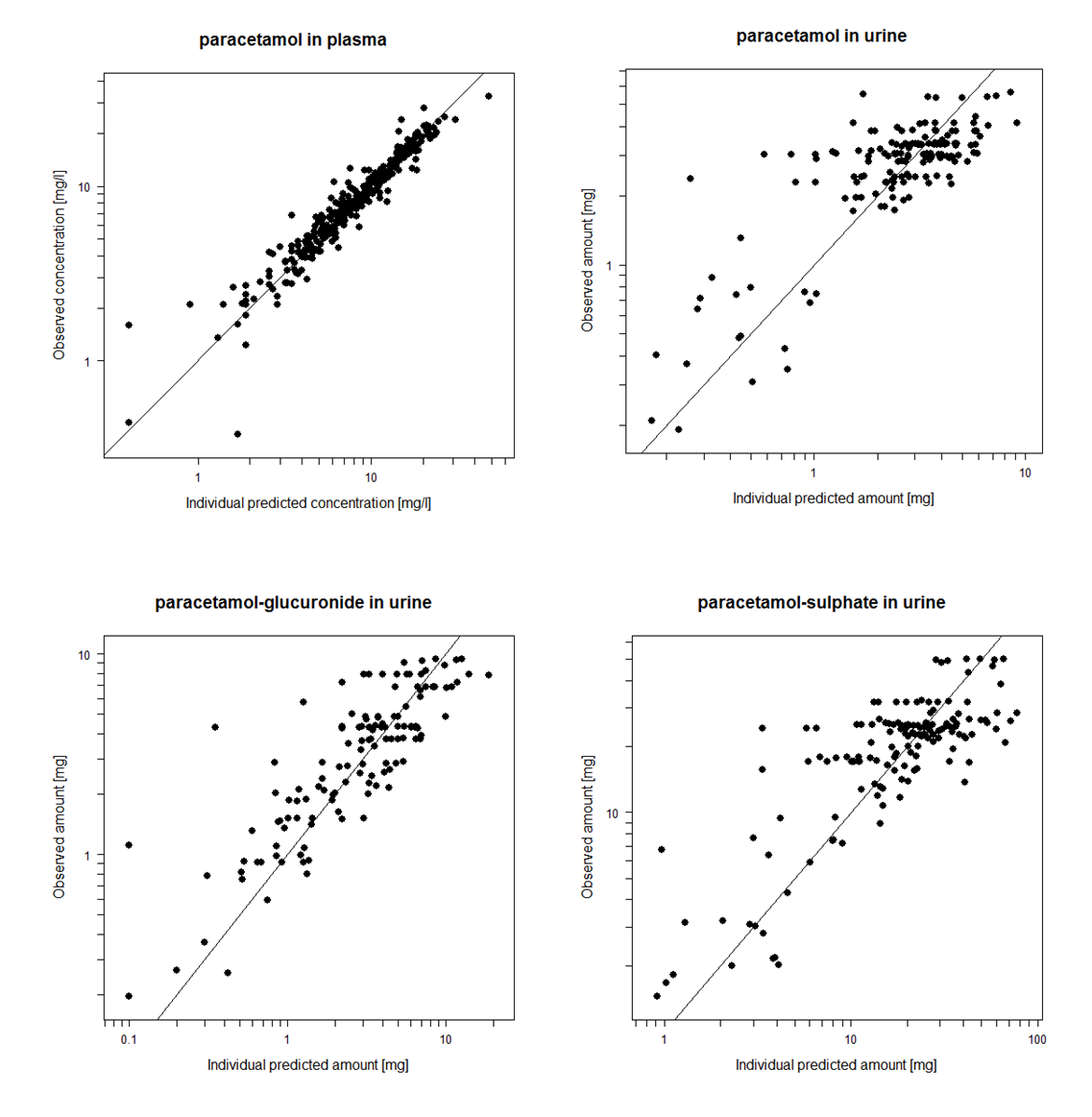

Supplement: Supplementary file 1 — Goodness-of-fit plots for the final model. a Individual predicted versus observed plots, b population predicted versus observed plots, c Conditional weighted residuals versus predicted concentration high resolution image (PNG 143 kb) [file 228_2015_1887_MOESM1_ESM.png]

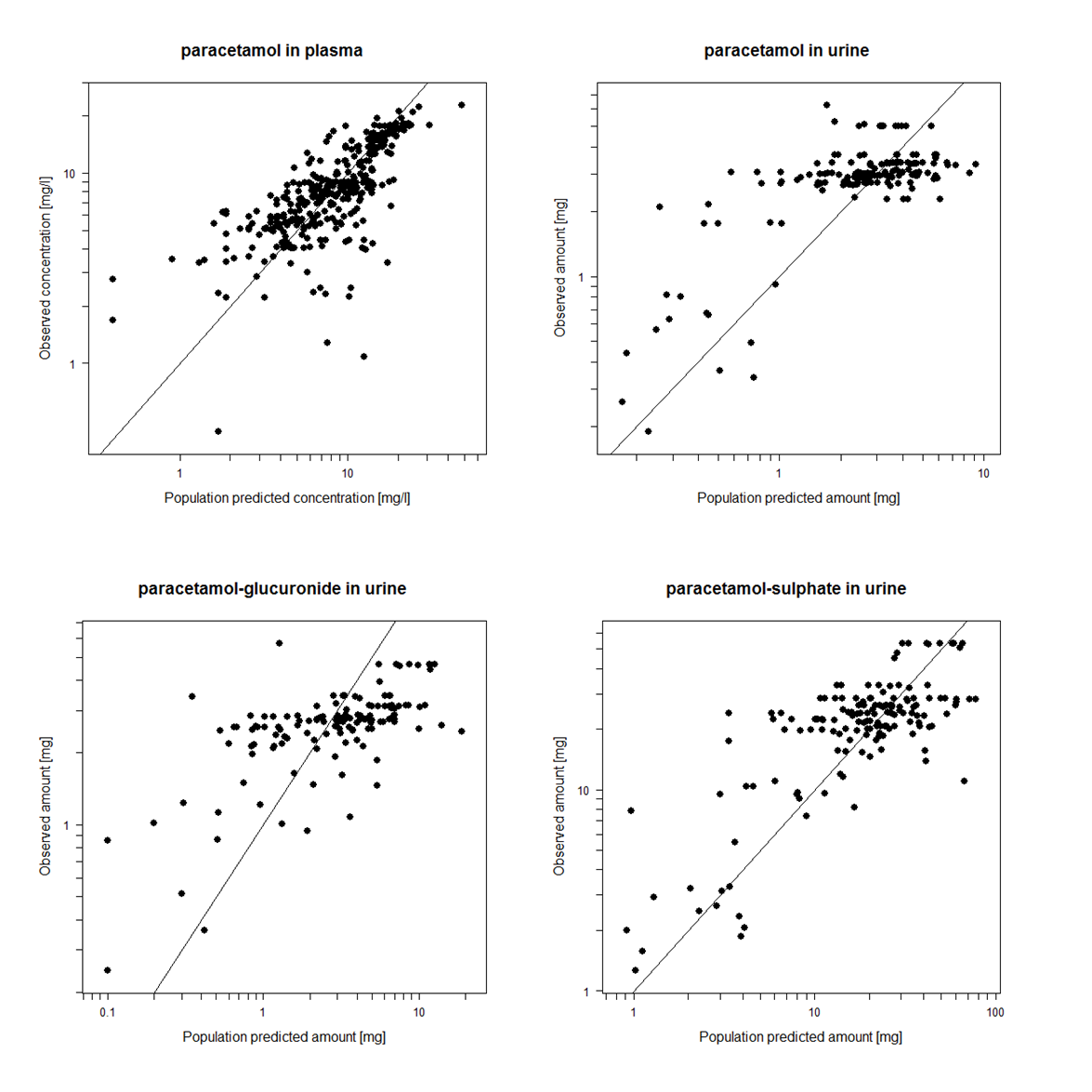

Supplement: Supplementary file 2 — (PNG 155 kb) [file 228_2015_1887_MOESM2_ESM.png]

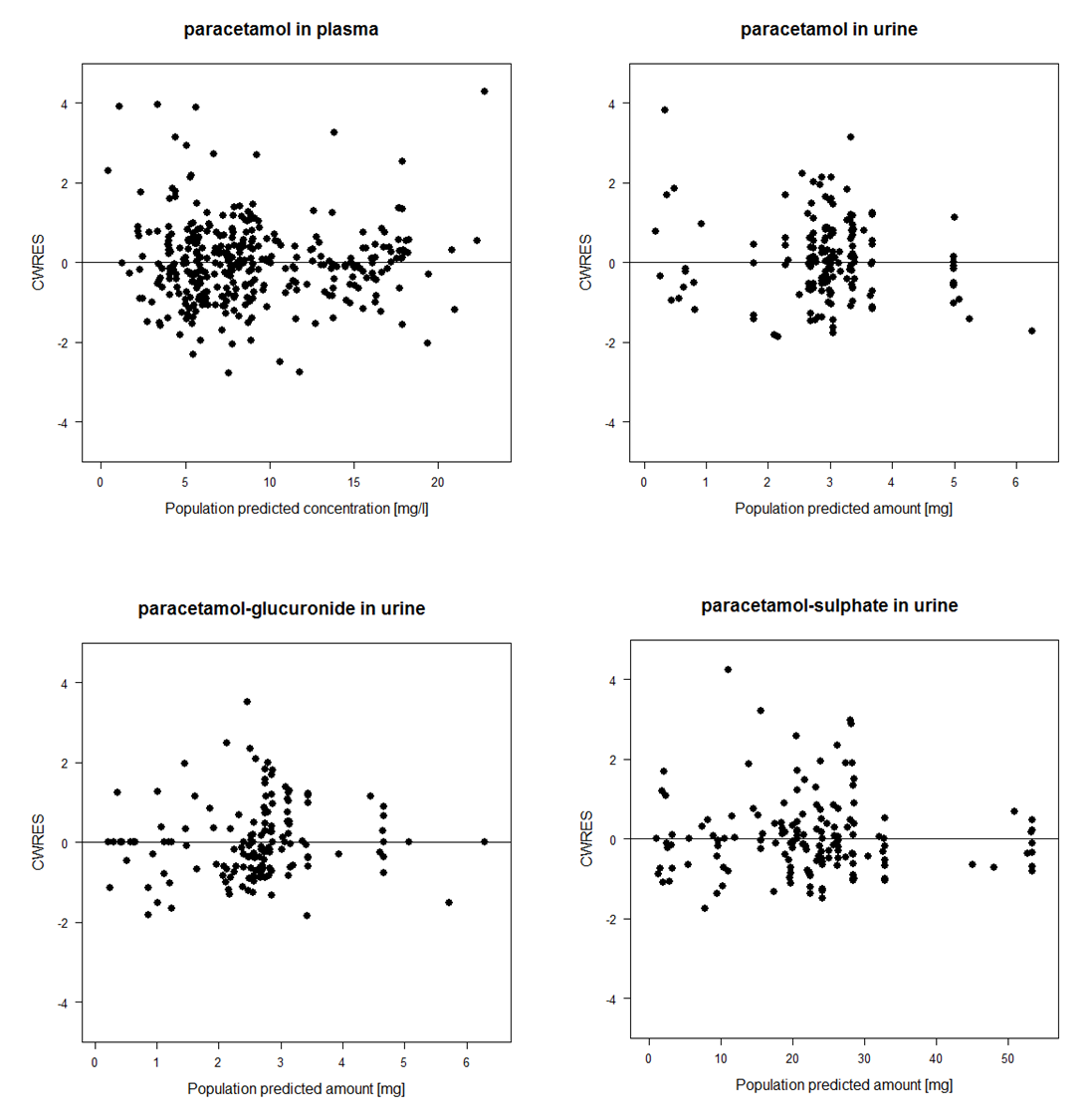

Supplement: Supplementary file 3 — High resolution image (TIFF 199 kb) [file 228_2015_1887_MOESM3_ESM.tif]

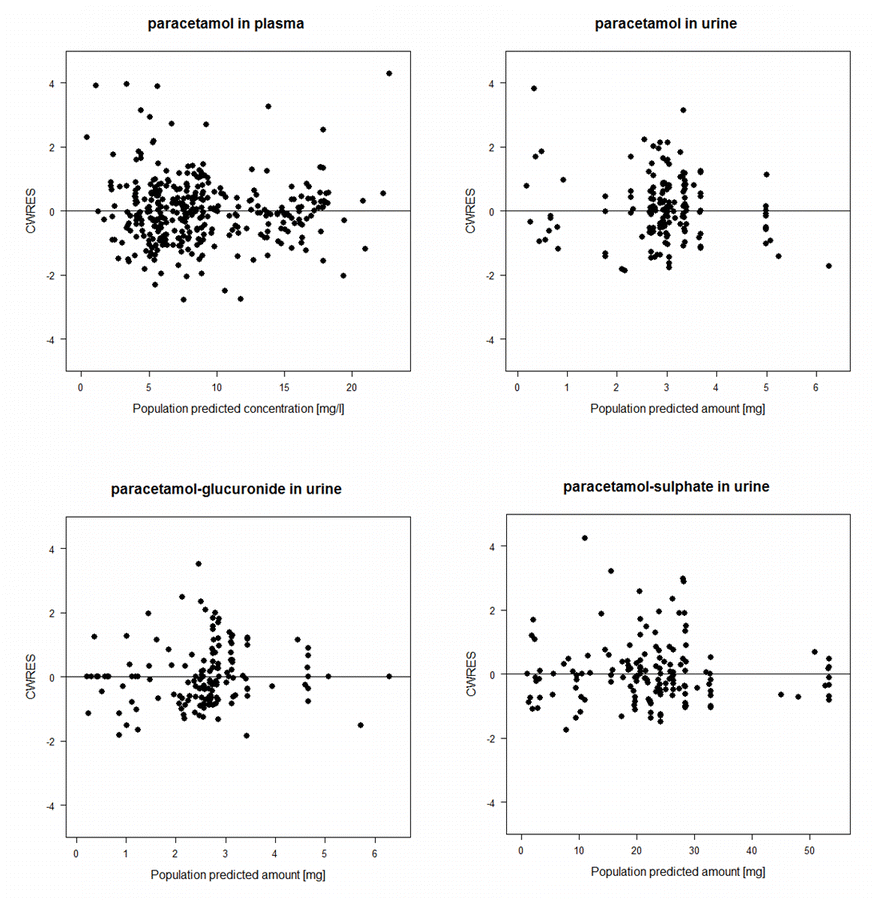

Supplement: Supplementary file 4 — (GIF 125 kb) [file 228_2015_1887_Fig4_ESM.gif]

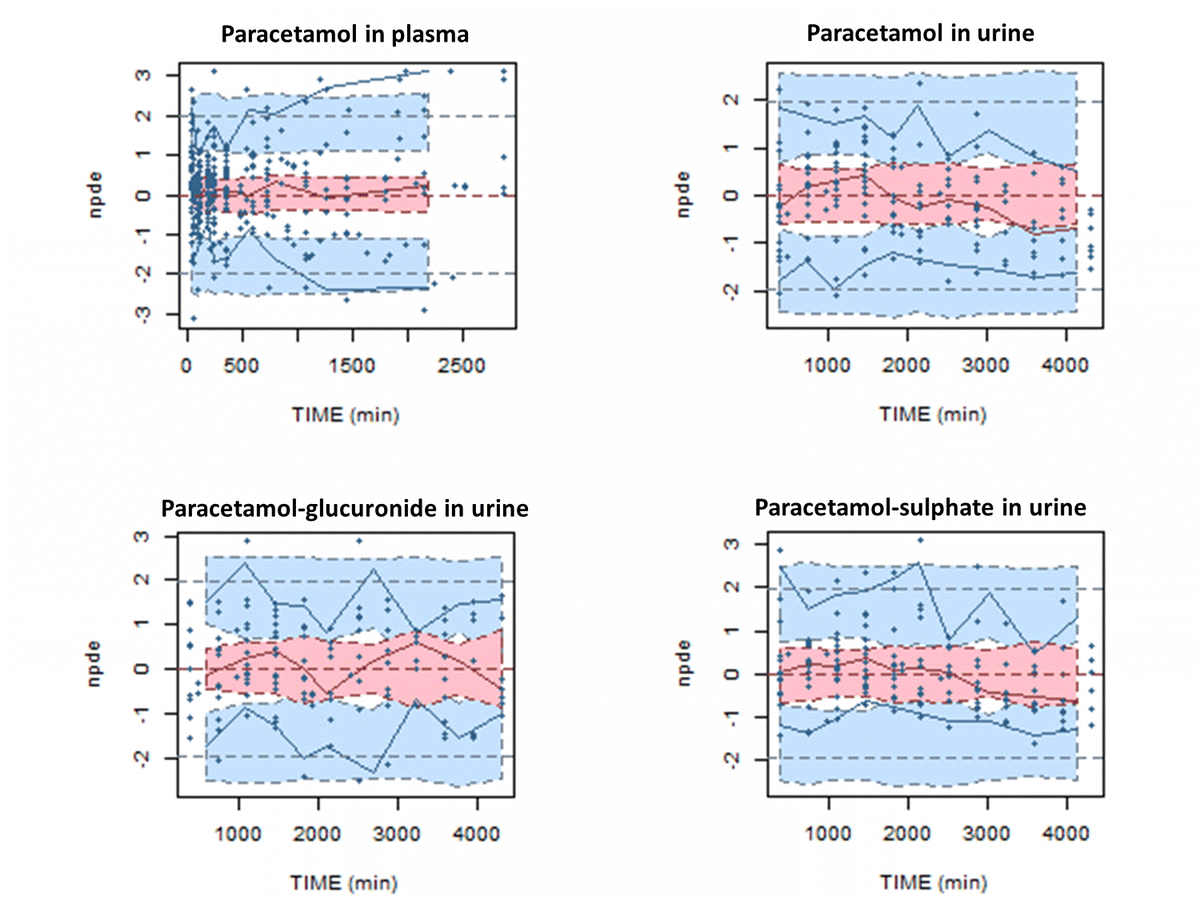

Supplement: Supplementary file 5 — Normalized prediction distribution errors versus time for paracetamol concentrations in plasma, and for the recovered amounts of paracetamol-glucuronide, unchanged paracetamol, and paracetamol-sulphate in urine high resolution image (GIF 266 kb) [file 228_2015_1887_Fig5_ESM.gif]

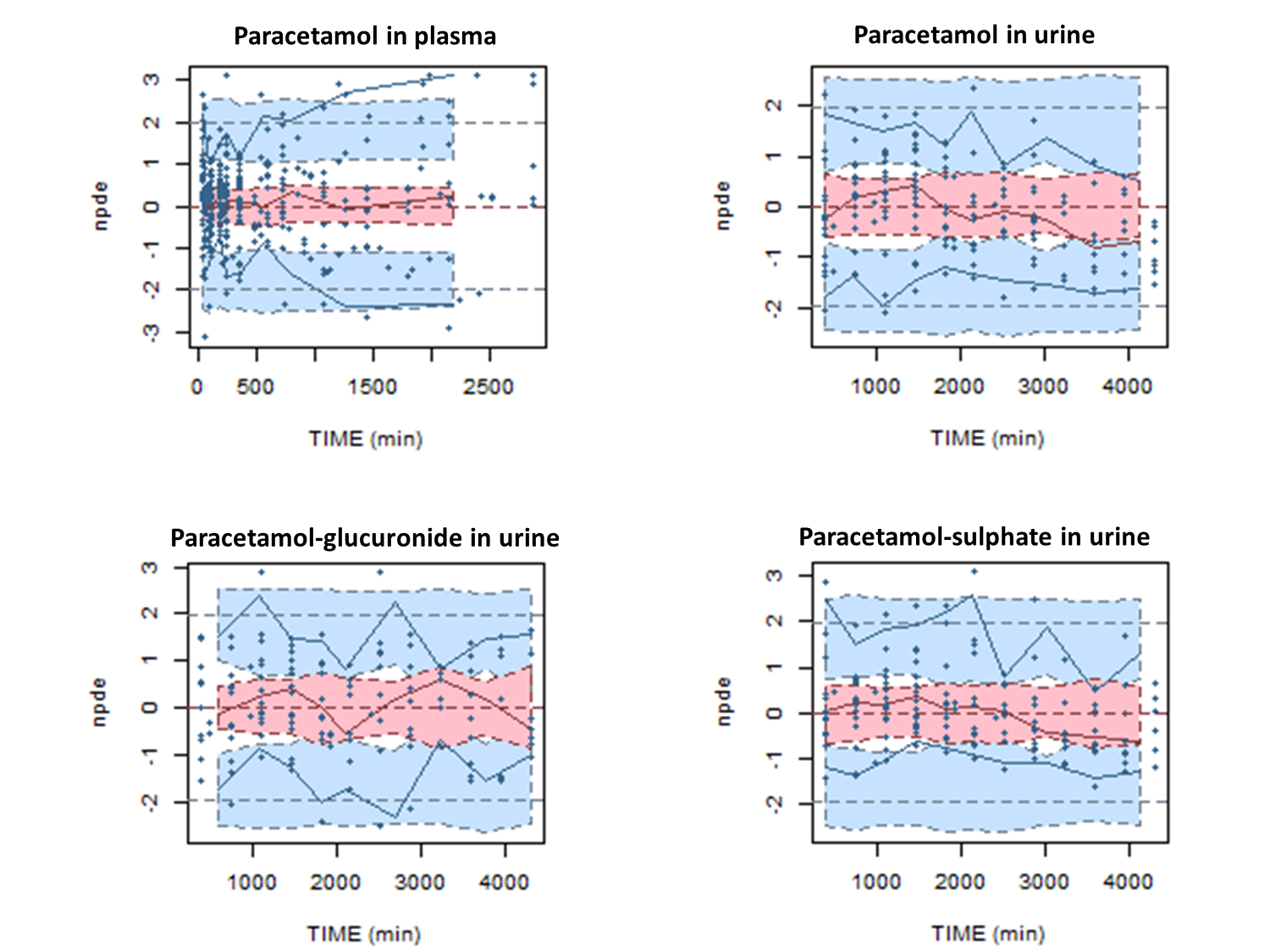

Supplement: Supplementary file 6 — High resolution image (TIFF 760 kb) [file 228_2015_1887_MOESM4_ESM.tif]
